# Disparities in the frequency of fruit and vegetable consumption by socio-demographic and lifestyle characteristics in Canada

**DOI:** 10.1186/1475-2891-10-118

**Published:** 2011-10-25

**Authors:** Sunday Azagba, Mesbah F Sharaf

**Affiliations:** 1 Department of Economics, Concordia University, 1455 de Maisonneuve Blvd. West, Montréal, Quebec, H3G 1M8, Canada

**Keywords:** fruit, vegetable, socio-demographic characteristics, lifestyle, quantile regression

## Abstract

**Background:**

The health benefits of adequate fruit and vegetable (F&V) consumption are significant and widely documented. However, many individuals self-report low F&V consumption frequency per day. This paper examines the disparities in the frequency of F&V consumption by socio-demographic and lifestyle characteristics.

**Method:**

This study uses a representative sample of 93,719 individuals from the Canadian Community Health Survey (2007). A quantile regression model is estimated in order to capture the differential effects of F&V determinants across the conditional distribution of F&V consumption.

**Results:**

The conditional and unconditional analyses reveal the existence of a socioeconomic gradient in F&V consumption frequency, in which the low income-education groups consume F&V less frequently than the high income-education groups. We also find significant disparities in F&V consumption frequency by demographic and lifestyle characteristics. The frequency of F&V consumption is relatively lower among: males, those in middle age, singles, smokers, individuals with weak social interaction and households with no children. The quantile regression results show that the association between F&V consumption frequency, and socio-demographic and lifestyle factors varies significantly along the conditional F&V consumption distribution. In particular, individual educational attainment is positively and significantly associated with F&V consumption frequency across different parts of the F&V distribution, while the income level matters only over the lower half of the distribution. F&V consumption follows a U-shaped pattern across the age categories. Those aged 30-39, 40-49 and 50-59 years consume F&V less frequently than those aged 18-29 years. The smallest F&V consumption is among the middle aged adults (40-49).

**Conclusions:**

Understanding the socio-demographic and lifestyle characteristics of individuals with low F&V consumption frequency could increase the effectiveness of policies aimed at promoting F&V consumption. The differential effects of individual characteristics along the F&V consumption distribution suggest the need for a multifaceted approach to address the variation in F&V consumption frequency.

## Introduction

The health benefits of fruit and vegetable (F&V) consumption are significant and widely documented [1,2]. According to reports from the World Health Organization and the Food and Agriculture Organization [3], daily consumption of five servings, or a minimum of 400 grams, of F&V helps in preventing several diseases. Several empirical studies document that a diet rich in F&V is negatively associated with the risks of: diabetes [4], obesity [5,6], strokes [7], high blood pressure [8]. Sufficient F&V consumption also helps in managing body weight because most F&V are high in water and fiber, and low in fat [5]. Globally, inadequate F&V consumption is responsible for annual deaths of 2.7 million, 11% of strokes, 31% of ischemic heart diseases and 19% of gastrointestinal cancers [3,9].

In spite of the numerous benefits of consuming F&V, many individuals self-report low F&V consumption frequency per day. For example, in 2010, 56.7% of Canadians aged 12 years and older reported consuming F&V less than five times a day [10], while in the U.S 67.5% of adults consume fruit less than two times per day and 73.7% consume vegetables less than three times per day [11].

Dietary choices including F&V consumption are largely affected by demographic factors like age and gender [12,13], psychological factors [14], socioeconomic class [15] and lifestyle behavior. Studies have shown that people of higher socioeconomic classes have healthier and nutritionally more balanced diets than those of lower socioeconomic classes [16-19]. Several studies find that, in terms of F&V consumption: men consume less than women [18,20-22], smokers consume less than non-smokers [21,22], and singles consume less than married people [16,18]. For example, Baker and Wardle [20] find that females consume more F&V than males, which they attribute to the poorer nutritional knowledge of males. The authors also find that males are less likely to know the recommended F&V intake, and the benefits associated with F&V consumption. Thompson et al. [22] find that individuals with low consumption of F&V are more likely to smoke, to be young and male.

Previous related studies mostly use standard multiple linear or binary choice regressions to estimate the determinants of the conditional mean of F&V consumption or the probability of consuming more than five servings of F&V a day. Results from these methods may be misleading insofar as individual F&V consumption responds differently to changes in the covariates at different regions of the F&V consumption distribution [23]. Multiple linear regressions treat different parts of the conditional distribution of F&V consumption equally and consider the marginal effect of the explanatory variables to be the same along the F&V consumption distribution.

This paper examines the socio-demographic and lifestyle determinants of F&V consumption frequency using quantile regression. Quantile regression allows the effect of each explanatory variable to vary along different percentiles of the conditional distribution of F&V consumption. Examining how individual socio-demographic and lifestyle factors influence the F&V consumption frequency at different consumption levels is particularly important in the nutrition literature where attention is given to the tails of the distribution.

## Methods

### Data

This study is based on a sample from the 2007 Canadian Community Health Survey (CCHS), a nationally representative, cross-sectional survey of 131,000 individuals of the Canadian population. It collects vital information on health-related behavior, as well as corresponding economic and socio-demographic variables. The survey excludes those living on Indian Reserves and Crown Lands, institutional residents, full-time members of the Canadian forces, and residents of certain remote regions, representing about 98% of the Canadian population aged 12 years and over. The sample of interest comprises of those aged 18-69 years which includes 93,719 individuals.

The frequency of F&V consumption, which is the dependent variable in this study, is the total number of times per day that a respondent consumes F&V. Statistics Canada derived total frequency of F&V consumption from a food frequency questionnaire. For a list of detailed survey questions and methods used, see Statistics Canada [24].

The study uses control variables that have been shown in previous studies to be important determinants of F&V consumption [e.g. [15-19]. Age is stratified into five categories: 18-29 (reference group), 30-39, 40-49, 50-59 and 60-69. Gender is represented by a dummy variable (male =0, female = 1). Marital status is represented by three dummy variables: married, separated and single (reference group). Four dummy variables are used to represent an individual's educational attainment: less than secondary (reference group), secondary, some post secondary, and post secondary. Household income is represented by four dummy variables: low income (reference group), low middle income, high middle income and high income. A dummy variable indicating individual social interaction (sense of belonging to a local community) is included (strong = 1, weak = 0). Smoking status is classified as: never smoker (reference group), current smoker, and former smoker. Immigration status is captured by a dummy variable (immigrant = 1, non-immigrant = 0). A dummy variable is used to indicate if a household has children, with having none as the reference group. In order to capture cultural or regional differences in F&V consumption, province fixed-effects are represented in five categories: Quebec (reference group), Ontario, British Colombia, Atlantic (comprising New Brunswick, Prince Edward Island, Nova Scotia and Newfoundland and Labrador) and Western (Alberta, Saskatchewan and Manitoba). A detailed definition of variables used in the study is presented in Table 1. The data used are the public-use-microdata version released by Statistics Canada, hence ethical approval is not required.

**Table 1:** Variables description and summary statistics

	Variables description	Mean	S.D
Fruits & vegetables	daily consumption of fruits and vegetables (frequency)	4.95	2.72
Age 18-29	age between 18 to 29	0.23	0.42
Age 30-39	age between 30 to 39	0.20	0.40
Age 40-49	age between 40 to 49	0.23	0.42
Age 50-59	age between 50 to 59	0.20	0.40
Age 60-69	age between 60 to 69	0.13	0.34
Male	gender is male	0.50	0.50
Female	Gender is female	0.50	0.50
Married	married/living with a partner/common-law	0.64	0.48
Separated	widowed/separated/divorced	0.10	0.30
Single	never married	0.25	0.43
Less secondary education	completed education is less than secondary	0.12	0.32
Secondary education	completed education is secondary	0.16	0.37
Some post secondary	completed education is some post secondary	0.09	0.28
Post secondary	completed education is post secondary	0.59	0.49
Low income	household income ( less than $30,000)	0.20	0.40
Low middle income	household income ($30,000-$49,999)	0.15	0.36
High middle income	household income ($50,000-$79,999)	0.14	0.35
High income	household income ($80,000 or more)	0.35	0.48
Strong social interaction	sense of belonging to community (strong)	0.60	0.49
Weak social interaction	sense of belonging to community (weak)	0.36	0.48
Have kids	household with kids	0.47	0.50
No kids	no kids in household	0.44	0.50
Current smoker	daily/occasional smoker	0.24	0.43
Former smoker	former daily/occasional smoker	0.38	0.48
Never smoker	never smoked	0.36	0.48
Immigrants	country of birth is not Canada	0.22	0.41
Non immigrants	country of birth is Canada	0.75	0.43
Quebec	province of residence is Quebec	0.23	0.42
Ontario	province of residence is Ontario	0.39	0.48
British Columbia	province of residence is British Columbia	0.13	0.34
Atlantic provinces	province of residence is New Brunswick, Prince Edward Island, Nova Scotia and Newfoundland and Labrador	0.07	0.25
Western provinces	province of residence is Alberta, Saskatchewan and Manitoba	0.16	0.37
*N*		93,719	

The statistics are weighted using the CCHS sampling weights.

### Statistical Analysis

To examine the disparities in F&V consumption frequency by socio-demographic and lifestyle factors at different points of the conditional F&V consumption distribution, the following quantile regression model is estimated:

(1) $$q_{\mu }\left(FV_{ij}|SES_{ij},X_{ij},\varphi_{j}\right)=\beta_{0}^{\mu }+SES_{ij}\beta_{1}^{\mu }+X_{ij}\beta_{2}^{\mu }+\varphi_{j}\beta_{3}^{\mu }$$

Where *q*_*μ *_represents the *μth *quantile of the conditional F&V consumption distribution. For example, *μ *= 50 is the conditional median estimate of F&V consumption. The subscript *i *stands for an individual and *j *for the corresponding province of residence. F&*V *denotes the daily frequency of fruit and vegetable consumption. *SES *denotes individual socioeconomic characteristics (education and income level). ***X ***is a vector of other control variables which includes: age, sex, marital status, immigration status, smoking status and social interaction. *φ *represents province fixed-effects, which capture regional and other cultural factors that may be associated with individual F&V consumption. For example, Quebec, a predominantly French speaking province, is a major F&V producer in Canada.

## Results

The summary statistics reported in Table 1 show that 59% of the sample has completed one or more years of post-secondary education and 12% have a less-than-secondary education. About 35% of the individuals live in a household with an annual income of more than $80,000, while 20% have household income of less than $30,000. 24% of the sample currently smokes, while 38% are former smokers. Half of the sample is male and 64% is married; 47% have children and 22% are immigrants.

**Figure 1:**
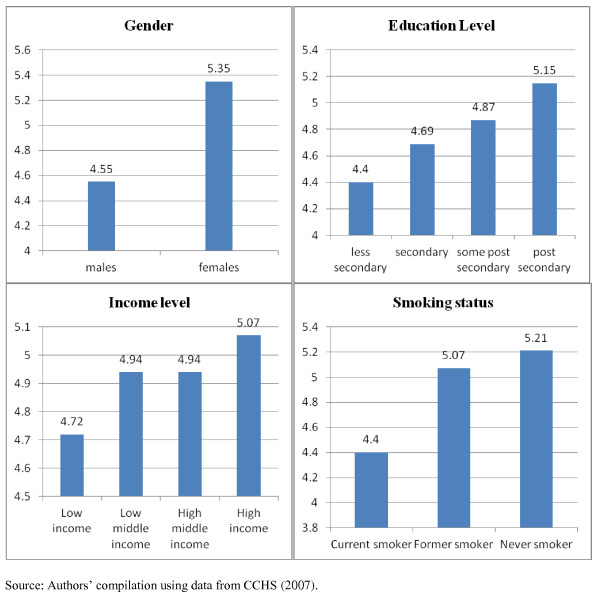
**Average daily consumption frequency of fruits and vegetables by selected characteristics**.

Table 1 shows that the average F&V consumption frequency is 4.95 per day. Although the population average implies high F&V consumption frequency, Figure 1 reveals wide disparities by socio-demographic and lifestyle characteristics. The standard deviation of 2.7 reported in Table 1 indicates a large variation in F&V consumption among individuals in the sample.

Figure 1 depicts the average daily F&V consumption frequency by selected characteristics. According to this unconditional analysis, the daily F&V consumption frequency is less for males and current smokers compared to females and never smokers respectively. The results also confirm the standard socioeconomic (SES) gradient in F&V consumption, where people with higher SES consume F&V more frequently than those with lower SES.

**Figure 2:**
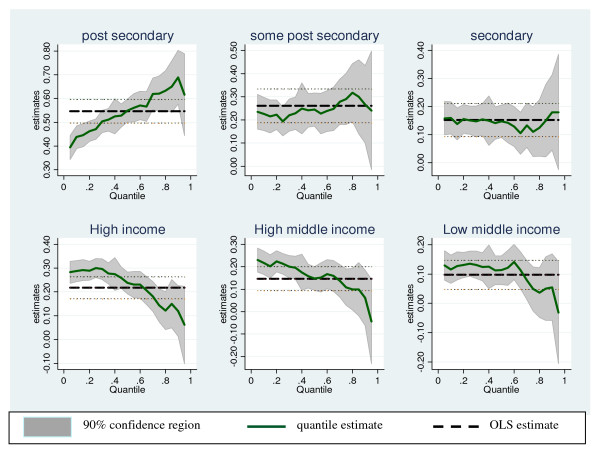
**Quantile regression estimates across conditional quantiles of the F&V distribution by socio-economic characteristics**.

Table 2 presents quantile regression estimates for some selected quantiles of the F&V distribution, as well as estimates for a baseline, ordinary least squares (OLS) model. The OLS and quantile regression results shown in Table 2 include the covariates described in the data section. Figure 2 summarizes the differences between the OLS and quantile estimates for socio-economic variables.

**Table 2:** Fruit and vegetable regression results: OLS and quantile estimates

	OLS	Quantile regression estimates					
	
		(5)	(15)	(25)	(50)	(75)	(90)
Less secondary (reference. group)							
post secondary	0.575***	0.369***	0.467***	0.490***	0.560***	0.678***	0.733***
	(0.044)	(0.041)	(0.042)	(0.042)	(0.041)	(0.057)	(0.107)
some post secondary	0.318***	0.243***	0.290***	0.232***	0.305***	0.426***	0.307**
	(0.066)	(0.067)	(0.056)	(0.059)	(0.060)	(0.085)	(0.154)
secondary	0.203***	0.185***	0.209***	0.173***	0.161***	0.139**	0.368***
	(0.057)	(0.048)	(0.047)	(0.049)	(0.049)	(0.070)	(0.138)
Low income (reference group)							
High income	0.129***	0.282***	0.242***	0.238***	0.190***	0.052	0.0212
	(0.041)	(0.037)	(0.034)	(0.037)	(0.037)	(0.053)	(0.010)
High middle income	0.033	0.157***	0.109***	0.079*	0.074*	-0.022	-0.040
	(0.049)	(0.044)	(0.041)	(0.045)	(0.045)	(0.062)	(0.118)
Low middle income	0.049	0.121***	0.104***	0.107***	0.083**	0.028	-0.025
	(0.048)	(0.041)	(0.037)	(0.040)	(0.042)	(0.060)	(0.113)
Age 18-29 (reference group)							
Age 30-39	-0.331***	0.0273	-0.122***	-0.185***	-0.287***	-0.359***	-0.664***
	(0.052)	(0.046)	(0.040)	(0.044)	(0.044)	(0.063)	(0.120)
Age 40-49	-0.412***	0.054	-0.105**	-0.186***	-0.382***	-0.509***	-0.849***
	(0.054)	(0.047)	(0.042)	(0.045)	(0.046)	(0.065)	(0.127)
Age 50-59	-0.307***	0.150***	0.007	-0.050	-0.263***	-0.437***	-0.766***
	(0.057)	(0.050)	(0.046)	(0.049)	(0.048)	(0.068)	(0.135)
Age 60-69	-0.083	0.382***	0.292***	0.219***	0.014	-0.200***	-0.679***
	(0.062)	(0.055)	(0.047)	(0.050)	(0.051)	(0.073)	(0.146)
Male (reference group)							
Female	0.763***	0.489***	0.574***	0.704***	0.828***	0.952***	0.986***
	(0.031)	(0.028)	(0.026)	(0.028)	(0.028)	(0.039)	(0.075)
Single (reference group)							
Married	0.156***	0.235***	0.271***	0.228***	0.235***	0.126**	-0.091
	(0.043)	(0.039)	(0.034)	(0.037)	(0.037)	(0.052)	(0.099)
Separated	0.003	-0.068	0.016	-0.045	0.022	0.011	-0.090
	(0.068)	(0.050)	(0.046)	(0.050)	(0.052)	(0.076)	(0.141)
Household with no kids (reference group)							
Household with kids	0.137***	0.086***	0.120***	0.128***	0.104***	0.157***	0.083
	(0.035)	(0.031)	(0.029)	(0.031)	(0.031)	(0.044)	(0.085)
Weak social interaction (reference group)							
Strong social interaction	0.379***	0.283***	0.309***	0.346***	0.414***	0.454***	0.374***
	(0.032)	(0.029)	(0.027)	(0.029)	(0.029)	(0.041)	(0.078)
Never smoker (reference group)							
Current smoker	-0.613***	-0.430***	-0.571***	-0.558***	-0.632***	-0.685***	-0.702***
	(0.042)	(0.036)	(0.034)	(0.036)	(0.037)	(0.053)	(0.010)
Former smoker	-0.082**	-0.037	-0.103***	-0.080**	-0.056*	-0.063	-0.102
	(0.036)	(0.033)	(0.030)	(0.033)	(0.033)	(0.046)	(0.087)
Canadian born (reference group)							
Immigrant	-0.044	0.031	0.021	0.013	0.023	-0.050	-0.093
	(0.042)	(0.042)	(0.037)	(0.040)	(0.040)	(0.056)	(0.105)
Quebec (reference group)							
Ontario	-0.741***	-0.266***	-0.341***	-0.433***	-0.749***	-1.000***	-1.200***
	(0.045)	(0.039)	(0.035)	(0.038)	(0.039)	(0.056)	(0.107)
British Columbia	-0.640***	-0.113**	-0.223***	-0.311***	-0.585***	-0.933***	-1.120***
	(0.052)	(0.051)	(0.044)	(0.047)	(0.047)	(0.067)	(0.126)
Atlantic	-1.109***	-0.467***	-0.699***	-0.797***	-1.057***	-1.413***	-1.512***
	(0.048)	(0.046)	(0.041)	(0.043)	(0.044)	(0.062)	(0.117)
Western	-0.712***	-0.314***	-0.424***	-0.525***	-0.729***	-0.937***	-1.059***
	(0.049)	(0.043)	(0.038)	(0.042)	(0.043)	(0.061)	(0.118)
Constant	4.702***	0.823***	1.822***	2.490***	4.084***	6.126***	8.717***
	(0.070)	(0.061)	(0.060)	(0.063)	(0.062)	(0.087)	(0.168)
							

Standard errors are in parentheses. *** p < 0.01, ** p < 0.05, * p < 0.1. The estimates are population weighted using the CCHS sampling weights.

The multivariate analyses which control for potential confounders are consistent with the descriptive statistics (see Figure 1) that females consume F&V more often than males. The OLS estimates show that on average, the daily F&V consumption frequency for females is 0.76 more than males. The quantile regression results show that this gender gap in F&V consumption frequency increases at higher quantiles on the conditional F&V distribution. Results show that smoking is significantly associated with low F&V consumption, where both current smokers and former smokers consume F&V less often than never smokers. On average, current smokers consume F&V less frequently compared to never smokers. This variation in F&V consumption frequency by smoking status is greater at higher percentiles of the conditional F&V distribution. We find that there is no statistically significant difference in F&V consumption between immigrants and natives. Also, results show that household composition significantly affects the frequency of F&V consumption. Married individuals and those with children consume F&V more often compared to their reference groups. Individuals with strong social interaction consume F&V more frequently than those with weaker social interaction.

F&V consumption frequency follows a U-shaped pattern across the age categories. Those aged 30-39, 40-49 and 50-59 years consume F&V less frequently than those aged 18-29 years. The smallest F&V consumption is among the middle aged adults (40-49).

The OLS results show no statistically significant difference in F&V consumption frequency between seniors (60-69) and the young (18-29), while the quantile estimates indicate a statistically significant difference. Seniors consume F&V more often than the young below the median. This pattern is reversed at the upper percentiles of the F&V consumption distribution. In line with the unconditional analysis, results from both the OLS and quantile regressions reveal the existence of a SES gradient in F&V consumption, where the low income-education groups consume F&V less often than the high income-education groups. The extent of this SES gradient varies across the conditional quantiles of the F&V consumption distribution. While individual educational attainment is positively and significantly associated with F&V consumption frequency across different parts of the F&V distribution, income level matters only at the lower half of the distribution. Figure 2 shows that the OLS model understates (overstates) the effect of income level on F&V consumption at the lower (higher) quantiles of the conditional F&V distribution. We find significant provincial differences in F&V consumption, where the Atlantic, Western, British Columbia and Ontario provinces consume F&V less often compared to the reference province (Quebec). The provincial effects are amplified at higher quantiles of the F&V consumption distribution.

## Discussion and Conclusion

In spite of the numerous health benefits from adequate consumption of F&V, the dietary behavior of many individuals with respect to F&V consumption is below the daily recommended level. A large and growing literature has examined the determinants of F&V consumption. Nonetheless, most previous studies are based on standard multiple linear or binary choice regressions. The findings from these estimation methods may lead to wrong policy intervention measures if individuals' F&V consumption responds differently to changes in the covariates at different regions of the F&V consumption distribution. Accordingly, we use a quantile regression to examine the disparities in F&V consumption frequency by socio-demographic and lifestyle characteristics along different parts of the F&V consumption distribution.

Both the conditional and unconditional analyses show significant disparities in F&V consumption frequency among people with different socio-demographic and lifestyle features. We find that F&V consumption is relatively lower among males, middle aged, singles, smokers, individuals with weak social interaction and households with no children. The results also reveal the existence of a SES gradient in F&V consumption where, low income-education groups consume F&V less often than the high income-education group. Estimates from the quantile regression show that socio-demographic and lifestyle factors exert different effects on F&V consumption frequency across the conditional quantiles of the F&V distribution. There is no statistically significant difference in F&V consumption between immigrants and natives. There are significant differences in F&V consumption between provinces, where the Atlantic, Western, British Columbia and Ontario provinces consume F&V less frequently compared to Quebec. This result could be due to cultural influence, since Quebec is a predominantly a French-speaking province. Quebec also has a long history of farming most notably in fruit, vegetable and dairy products.

Several explanations have been used in the literature to justify the disparities in F&V consumption by socio-demographic characteristics [e.g. [16-22]]. For example, it has been suggested that educational attainment affects nutritional knowledge and awareness about the risks associated with inadequate consumption of F&V. One potential explanation for the disparities in F&V consumption by income level is due to the high price of F&V. The difference in F&V consumption by marital status may be due to family or household size, where individuals tend to consume more F&V when eating meals with others [17].

The findings of this paper are consistent with several previous studies which find that men consume less F&V than women [18,20-22], smokers consume less than non-smokers [21,22], singles consume less than married people [16,18] and that there is no significant difference by ethnicity [16,25]. The existence of a socioeconomic gradient in F&V consumption is in line with the findings of several studies which find a positive association between income, level of education and F&V consumption [16-19]

The current study has some limitations. First, the cross-sectional design of the data set limits ability to infer causality and does not allow us to control for unobserved factors that may affect the consumption of F&V, such as preferences. This calls for further research using longitudinal data. Second, due to data set limitations, F&V consumption data are based on a survey question that measures the number of times daily, respondents reported that they consumed F&V. This F&V consumption frequency may not reflect the actual quantity consumed [10].

Understanding the socio-demographic and lifestyle characteristics of individuals with low F&V consumption frequency helps to identify the targeted groups for nutrition promotion policies aimed at encouraging F&V consumption. Intervention measures need to take into account the potential heterogeneous effect of F&V consumption determinants along the different quantiles of the F&V distribution. There is no one-size-fits-all strategy to promote healthy eating behavior; a multifaceted approach would be required to address low consumption of F&V successfully. For example, increasing people's awareness about the benefits of F&V consumption, through the media and other community-organized nutrition programs, as well as subsidizing the cost of F&V may be helpful in encouraging the consumption of F&V, especially among people in low socioeconomic strata.

## Competing interests

The authors declare that they have no competing interests.

## Authors' contributions

Both Authors contributed equally to the conceptualization, design and composition of the paper.

All authors read and approved the final manuscript.
